# Thrombospondin 1 Deficiency Ameliorates the Development of Adriamycin-Induced Proteinuric Kidney Disease

**DOI:** 10.1371/journal.pone.0156144

**Published:** 2016-05-19

**Authors:** Hasiyeti Maimaitiyiming, Qi Zhou, Shuxia Wang

**Affiliations:** 1 Department of Pharmacology and Nutritional Sciences, University of Kentucky, Lexington, Kentucky, United States of America; 2 Lexington Veterans Affairs Medical Center, Lexington, Kentucky, United States of America; University of Utah School of Medicine, UNITED STATES

## Abstract

Accumulating evidence suggests that thrombospondin 1 (TSP1) is an important player in diabetic nephropathy. However, the role of TSP1 in podocyte injury and the development of non-diabetic proteinuric kidney disease is largely unknown. In the current study, by using a well-established podocyte injury model (adriamycin-induced nephropathy mouse model), we examined the contribution of TSP1 to the development of proteinuric kidney disease. We found that TSP1 was up-regulated in the glomeruli, notably in podocytes, in adriamycin injected mice before the onset of proteinuria. ADR treatment also stimulated TSP1 expression in cultured human podocytes *in vitro*. Moreover, increased TSP1 mediated ADR-induced podocyte apoptosis and actin cytoskeleton disorganization. This TSP1’s effect was through a CD36-dependent mechanism and involved in the stimulation of p38MAPK pathway. Importantly, in vivo data demonstrated that TSP1 deficiency protected mice from ADR induced podocyte loss and foot process effacement. ADR induced proteinuria, glomerulosclerosis, renal macrophage infiltration and inflammation was also attenuated in TSP1 deficient mice. Taken together, these studies provide new evidence that TSP1 contributes to the development of non-diabetic proteinuric kidney disease by stimulating podocyte injury and the progression of renal inflammation.

## Introduction

Thrombospondin1 (TSP1) belongs to a family of five secreted glycoproteins encoded by separated genes. It is released from activated platelet and is also synthesized and secreted by many cell types including endothelial cells, smooth muscle cells, kidney mesangial cells, tubular cells, and podocytes et al. [1]. TSP1 is a 420–450 kDa homotrimer with individual subunits of approximately 145 kDa. It is composed of several domains that can interact with specific cell surface receptors/molecules to modulate cell adhesion, growth, motility, differentiation, and survival [2–7]. Recently, accumulating evidence suggests that TSP1 is an important player in a variety of kidney diseases such as ischemia-reperfusion induced kidney injury and obesity/diabetes associate nephropathy [8–15]. TSP1 has been identified to be an important physiological activator for latent TGF-β activation [16,17]. Previous studies from our lab and others demonstrated that glomerular mesangial cells produced excessive amount of TSP1 in response to diabetic stimuli, which stimulated the profibrotic factor-TGF-β activation and the development of glomerulosclerosis [8,10,12,13,18–22]. Although the effect of TSP1 on mesangial cell function has been extensively studied, a role for TSP1 in regulating podocyte function and its contribution to non-diabetic proteinuric kidney disease has not been explored.

Podocytes are highly differentiated cells that play a critical role in maintaining glomerular infiltration barrier. Podocyte dysfunction, injury or loss leads to a broad spectrum of clinical syndromes such as diffuse mesangial sclerosis, focal segmental glomerulosclerosis (FSGS), and diabetic nephropathy (DN) et al [23]. There is evidence that injured podocytes expressed more TSP1 as determined by in situ hybridization of specimens from patients with primary FSGS [24]. Moreover, in type 1 diabetic mouse model, global TSP1 deficiency or TSP1 antagonist preserved podocyte marker expression (e.g. nephrin) and attenuated albuminuria [18,21]. In addition, in a high fat diet-induced obesity-related nephropathy mouse model in our previous studies, global TSP1 deficiency protected podocyte from FFA induced apoptosis and attenuated albuminuria [8,25]. Together, all of these *in vivo* animal and human data support the important role of TSP1 in podocyte injury and the development of proteinuric kidney disease.

In the current study, we utilized a well-established podocyte injury and experimental FSGS animal model: adriamycin (ADR) induced nephropathy mouse model. The rationale for choosing this ADR-nephropathy model to study TSP1’s effect is based on a human study showing that podocyte TSP1 was significantly elevated in patients with primary FSGS [24]. Therefore, by using this model, the contribution of TSP1 to the podocyte injury and the development of proteinuric kidney disease *in vitro* as well as *in vivo* was examined.

## Materials and Methods

### Experimental animals and protocols

All experiments involving animals conformed to the National Institutes of Health Guide for the Care and Use of Laboratory Animals and were approved by the University of Kentucky Institutional Animal Care and Use Committee. Eight week old male TSP1 -/- mice and wild type (WT) controls (on C57BL6/J background, maintained colony by our lab) were used in this study. Considering the relative resistance of B6 strain to Adriamycin (ADR) induced nephropathy [26], a single high dose of ADR (19 mg/kg) was administered into TSP1-/- and WT mice through tail vein [27–29]. After two weeks of ADR injection, mice underwent 24 h urine collection in metabolic cages and then sacrificed by Carbon Dioxide asphyxiation and followed by cervical dislocation. Kidneys were harvested for further analysis. In another set of experiment, we used BALB/c mice to determine how glomerular TSP1 expression was altered in ADR-induced nephropathy. BALB/c mouse has been identified to be a susceptible strain to ADR induced nephropathy [30,31]. Eight week old male BALB/c mice was injected with a single dose of ADR at 11 mg/kg body weight by tail vein as previously described [32]. After 3, 7, or 14 days of injection, mice were sacrificed and kidneys were harvested for further analysis. Saline injection was used as control. Each time points contained 3–5 mice. In these experiments, animals with more than 20% weight loss were sacrificed by Carbon Dioxide asphyxiation (followed by cervical dislocation) and excluded from the study (humane endpoints).

### Renal function, immunohistochemical or immunofluorescence staining, and electromicroscopy

Urinary albumin and creatinine were determined by using mouse albumin ELISA and creatinine companion kits (Philadelphia, PA USA) following manufacturer’s protocols. Serum levels of creatinine were determined by using a kit from Biovision (Milpitas, CA). For immunohistochemical staining, formalin-fixed and paraffin-embedded renal tissues were cut into 4- to 5-μm sections. The slides were stained with anti-collagen IV (Cell Signaling) or F4/80 antibody (Serotec) using VECTASTAIN Elite ABC system (Vector Lab) as described previously [25]. For immunofluorescence staining: frozen kidney samples were sectioned and fixed in cold acetone. The slides were stained with anti-WT1 antibody (Novus) and then with fluorescence conjugated secondary antibodies (Life Technologies). After washing, slides were incubated with DAPI for 5 minutes and coverslipped. In addition, kidney electron microscopy (EM) was performed by using the service from the Imaging Center at University of Kentucky.

### Western blotting analysis

Glomeruli were isolated from mice by gradually sieving method as previously described [33]. Briefly, kidneys were minced and digested in collagenase A (Sigma) at 37°C for 30 minutes with gentle agitation. The digested kidney tissues were gently pressed through a 100-μm cell strainer. The filtered tissues were then passed through 70 μm and 40 μm cell strainer. The final filtered tissues were collected and centrifuged at 2,000 rpm for 5 minutes. The pellet enriched in glomeruli was collected. Mouse glomeruli and podocytes were homogenized. Equal amount of total protein was subjected to SDS-PAGE gel under reducing conditions and transferred onto a nitrocellulose membrane. After blocking, the membrane was incubated with anti-TSP1 (Thermo Scientific), anti-CD36 (Novua), anti-phospho-p38 and anti-total p38 antibodies (Cell Signaling) and then with horseradish peroxidase-conjugated secondary antibody (Jackson Labs). The reaction was visualized using an enhanced chemiluminescence system (Pierce). Immunoblots were analyzed by scanning densitometry and quantified by Quantity One gel Analysis software (Bio-Rad Laboratories).

### Real-time PCR

Total RNA was isolated from glomeruli or podocytes using TRIzol reagent (Invitrogen) and treated with DNaseI (Roche). The treated RNA was cleaned up using an RNeasy kit (Qiagen). Two micrograms of total RNA was used for cDNA synthesis with a High Capacity cDNA Reverse Transcription kit (Invitrogen). Real-time PCR analyses were performed using a SYBR Green PCR Master Mix kit with a MyiQ Real-time PCR Thermal Cycler (Bio-Rad Laboratories). The quantities of each test gene and internal control 18S RNA were then determined from the standard curve using MyiQ system software, and mRNA expression levels of test genes were normalized to 18S RNA levels as described previously. The primer sequences were provided in Table 1.

**Table 1:** Primer sequences for q-PCR.

	FORWARD	REVERSE
**mCD11c**	CTGGATAGCCTTTCTTCTGCTG	GCACACTGTGTCCGAACTC
**mF4/80**	CTTTGGCTATGGGCTTCCAGTC	GCAAGGAGGACAGAGTTTATCGTG
**mIL-1β**	TGGAGAGTGTGGATCCCAAGCAAT	TGTCCTGACCACTGTTGTTTCCCA
**mCollagen IV**	AGG GTTCCCAGGTTCTAA	GCCCAACGTCACCTTTAT
**mTNF-α**	AGCCGATGGGTTGTACC	TGAGT GGTCCCCCTTCT
**mMCP-1**	CAGCCAGATGCAGTTAACGC	GCCTACTCATTGGGATCATCTTG
**hTSP1**	CCGCCGATTCCAGATGATTCCT	ACGAGTTCTTTACCCTGATGGCGT
**hCD36**	CCCTGTTACTACCACAGTTG	ATGTCGCAGTGACTTTCC
**mCD31**	ACACTATGTGGACTGGCAGTGGTT	TGAGGCTCGATTGTTCAGCTGCTA

### Human podocyte culture and treatments

Immortalized human podocytes (generously provided by Dr. Moin Saleem from Bristol Royal Hospital for Children, Bristol, UK) were cultured and differentiated as described previously [25,34]. Differentiated podocytes were treated with ADR (Sigma, 20μg/ml) for different time periods. After treatment, 1). Cells were harvested and mRNA levels of TSP1 and CD36 were determined by real-time PCR. 2). Podocyte apoptosis was determined by a transferase-mediated dUTP nick-end labeling (TUNEL) staining of podocytes according to the manufacturer's instructions (Roche). Caspase 3 activity in cell lysate was measured by using caspase-3 colorimetric assay kit (R&D) as described previously [25]; 3). For F-actin Staining: cells were grown on four-well chambers. After overnight treatment with ADR, cells were fixed in 4% paraformaldehyde solution, permeablized with 0.1% Triton X-100, blocked in 1% BSA/PBS and stained with Texas Red-Phalloidin (Lifetech). After washing, the slides were sealed with DAPI mounting medium and visualized under fluorescence microscope. In addition to ADR treatment, differentiated human podocytes were treated with recombinant human TSP1 (R&D system) at 0.1–5 μg/ml in the presence or absence of anti-CD36 antibody (10–15 ug/ml, clone FA6-152 IgG1 from Abcam) or SB202190 (p38 inhibitor, at 10^−5^ mol/L, Sigma) for indicated time periods. After treatment, cell apoptosis assay and F-actin staining were performed as described above. The levels of phospho-p38MAPK and total p38 in cell lysates were determined by immunoblotting.

### Isolation of murine podocytes and treatments

Podocytes from TSP1 knockout mice or control wild type mice were isolated using the protocol as previously described [25,35,36]. The purity of isolated podocytes was determined by staining with the podocyte marker WT1 or by determining WT1 mRNA levels by real-time PCR. Podocytes of passages 1 or 2 were used for the experiments. Podocytes were treated with ADR (20 μg/ml) for 24 hours. After treatment, 1) Cell apoptosis was determined by TUNEL assay. 2) Caspase 3 activity in cell lysates was analyzed by using caspase-3 colorimetric assay kit (R&D). 3) Cells were stained with Texas Red-Phalloidin to examine the actin cytoskeleton organization as described above.

### Statistical analysis

Data are the mean ± SEM. Differences between groups were determined by two-way ANOVA or one-way ANOVA followed by Turkey's post hoc tests or Student's t-test as appropriate. The significance level was *P* <0.05.

## Results

### Expression of TSP1 and its receptor-CD36 expression was up-regulated in podocytes from adriamycin (ADR) treatment in vivo as well as in vitro

**Fig 1:**
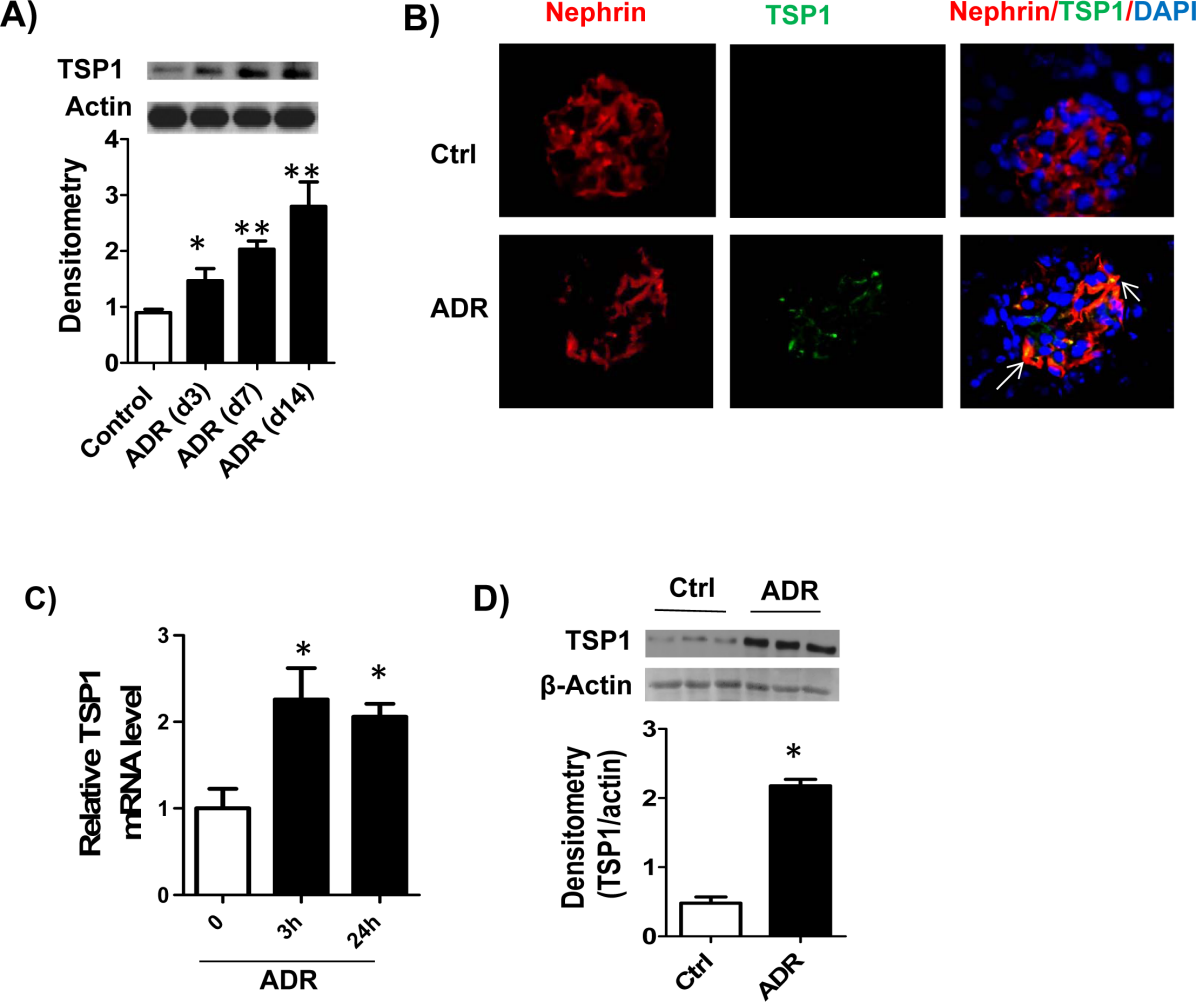
TSP1 expression was up-regulated in podocytes from adriamycin (ADR) treatment *in vivo* as well as *in vitro*. (A). Glomeruli TSP1 expression from ADR injected BALB/c mice was determined by immunoblotting. Data are represented as mean ± SE (n = 3 mice /group). * p<0.05, ** P<0.01 vs. control. (B). Colocolization of TSP1 with nephrin in glomeruli was determined by immunofluorescence staining of kidney samples from ADR injected mice. ADR (20μg/ml) treated human podocyte was harvested for analyzing TSP1 expression in mRNA (C) and protein (D) levels by real-time PCR and immunoblotting, respectively. Data are presented as mean ± SE (n = 3 individual experiments).

To address the role of TSP1 in podocyte injury and the development of proteinuric kidney disease, first, we studied its expression in glomerulus from experimental FSGS model-ADR induced nephropathy. Under normal conditions, immunoblot analysis of mouse glomerular lysates showed minimal TSP1 expression. TSP1 expression was markedly increased in the glomeruli lysates from ADR injected BALB/c mice as early as three days after ADR injection, a time point without onset of proteinuria [30–32] and continued to increase till the end of the study (day 14) (**Fig 1A**). In addition, double immunofluorescence staining data showed that increased expression of TSP1 in the glomeruli was mostly colocolized with podocyte marker-nephrin (**Fig 1B**). This data is consistent with studies from primary human FSGS kidney samples showing that injured podocytes expressed more TSP1 in glomeruli [24], suggesting that podocyte is a cellular source of increased glomerular TSP1 in both human and experimental FSGS models. This is further supported by *in vitro* data showing that ADR treatment significantly induced TSP1 expression in cultured human podocytes (**Fig 1C and 1D)**. However, ADR treatment had no effect on TSP1 expression in mesangial cells or glomerular endothelial cells (data not shown).

**Fig 2:**
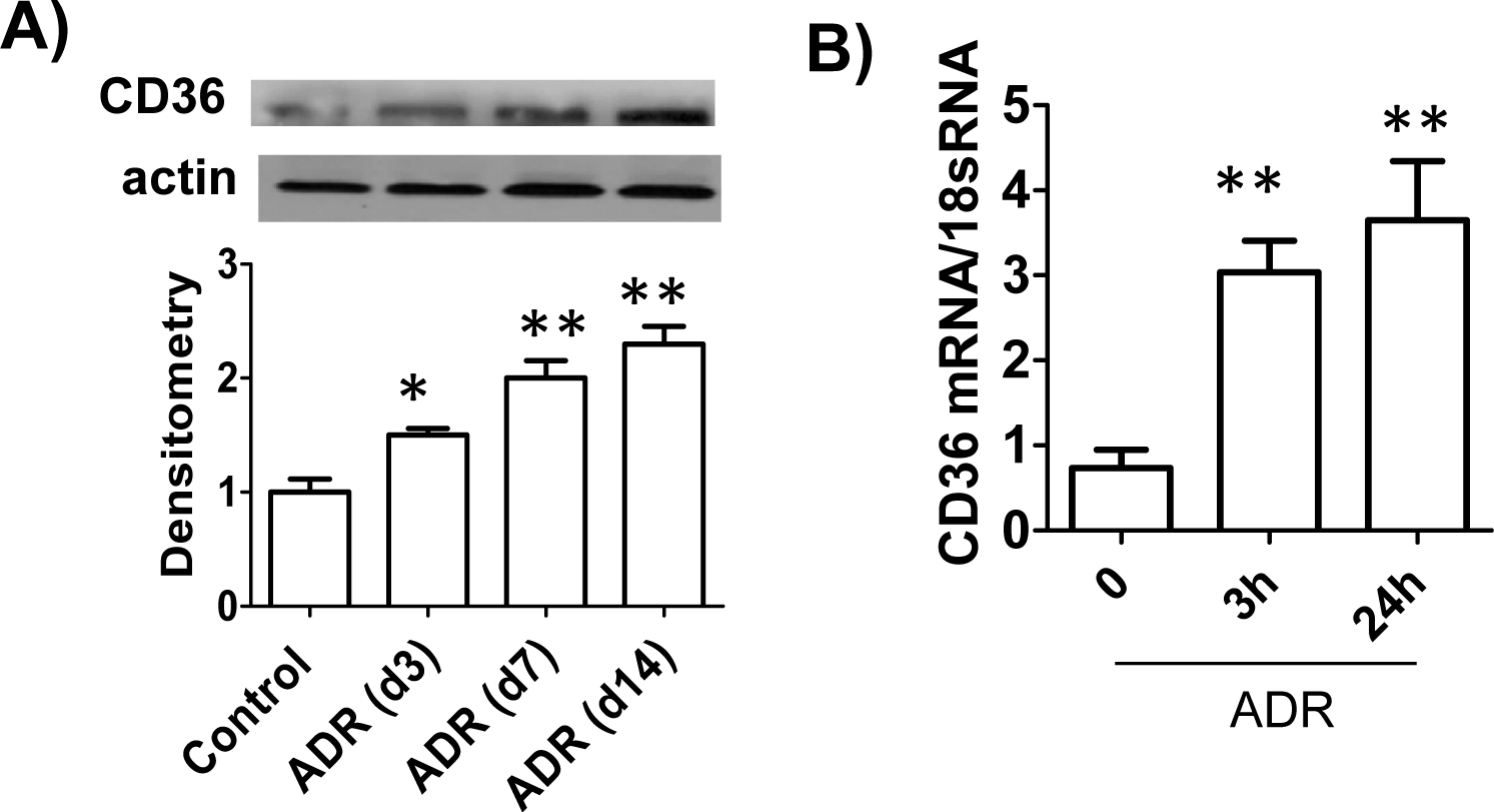
CD36 expression was up-regulated in podocytes from ADR treatment *in vivo* as well as *in vitro*. (A). Glomeruli CD36 expression from ADR injected BALB/c mice was determined by immunoblotting. Data are represented as mean ± SE (n = 3 mice /group). * p<0.05, ** P<0.01 vs. control. (B). ADR (20μg/ml) treated human podocyte was harvested for analyzing CD36 expression by real-time PCR. Data are presented as mean ± SE (n = 3 individual experiments). ** P<0.01 vs. 0h.

Next, we determined the expression of CD36 (one of TSP1’s receptors on podocytes) in this model. Similar to the TSP1 levels, CD36 expression was also upregulated in the glomeruli from ADR injected BALB/c mice or in ADR treated human podocytes (**Fig 2**). Taken together, these results indicated that expression of TSP1 and its receptor-CD36 was up-regulated in podocytes from ADR treatment in vivo as well as in vitro.

### ADR or TSP1 treatment induced podocyte injury through a CD36-dependent activation of p38MAPK pathway

**Fig 3:**
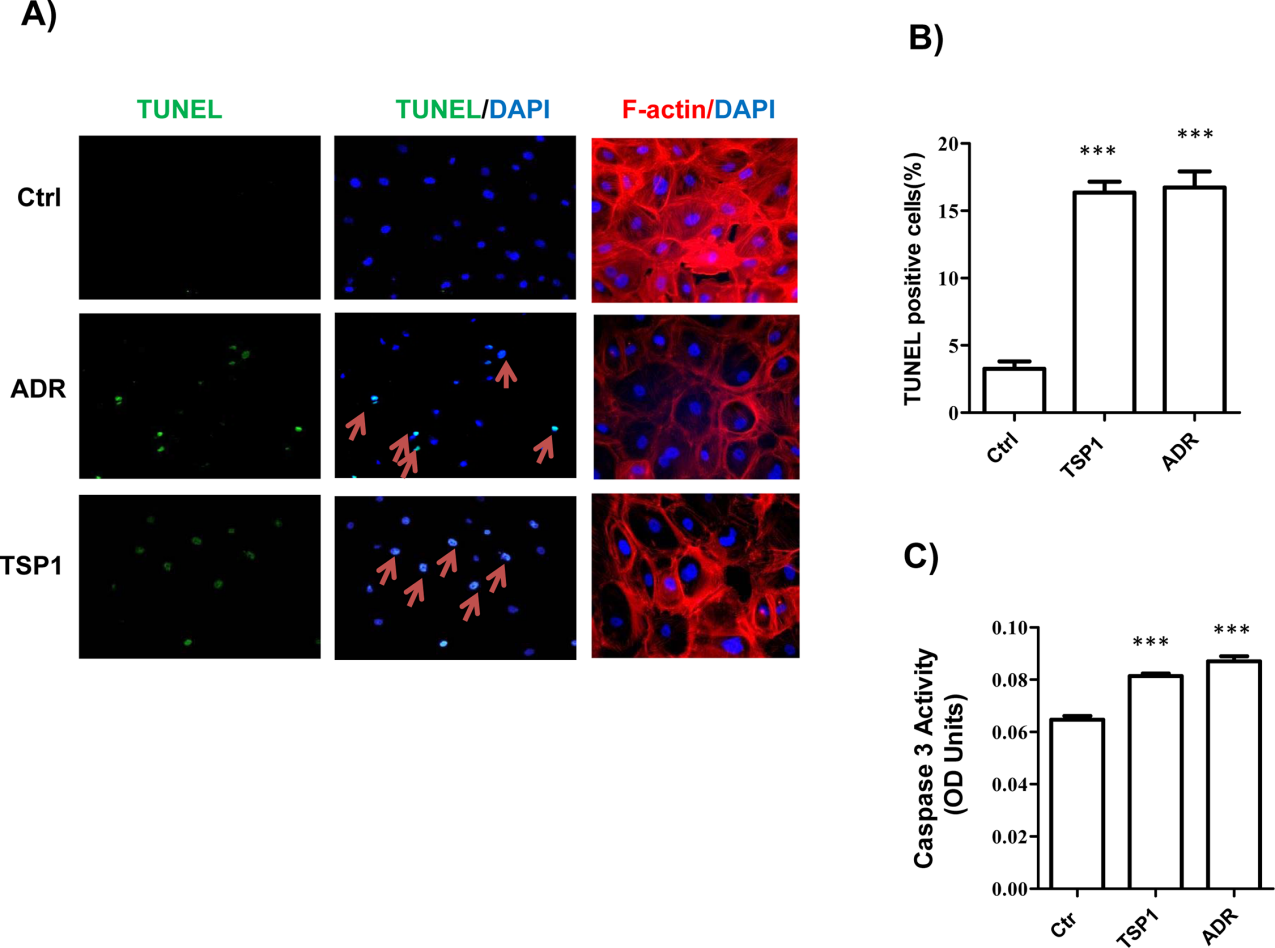
ADR or TSP1 treatment induced human podocyte apoptosis and actin cytoskeleton disorganization. Human podopcytes were treated with ADR (20μg/ml) or TSP1 (5μg/ml) for 24 hours. Then, TUNEL and F-actin staining was performed (A). TUNEL positive cells were calculated (B). Caspase 3 activity in cell lysates was measured (C). Data are presented as mean ± SE (n = 3 individual experiments). ***, *P* <0.001 vs. control.

We determined the effect of ADR or TSP1 treatment on human podoyte injury *in vitro*. As shown in **Fig 3**, ADR or TSP1 treatment induced podocyte actin cytoskeletal disorganization (determined by F-actin staining) and apoptosis (determined by TUNEL staining and Caspase 3 activity measurement).

**Fig 4:**
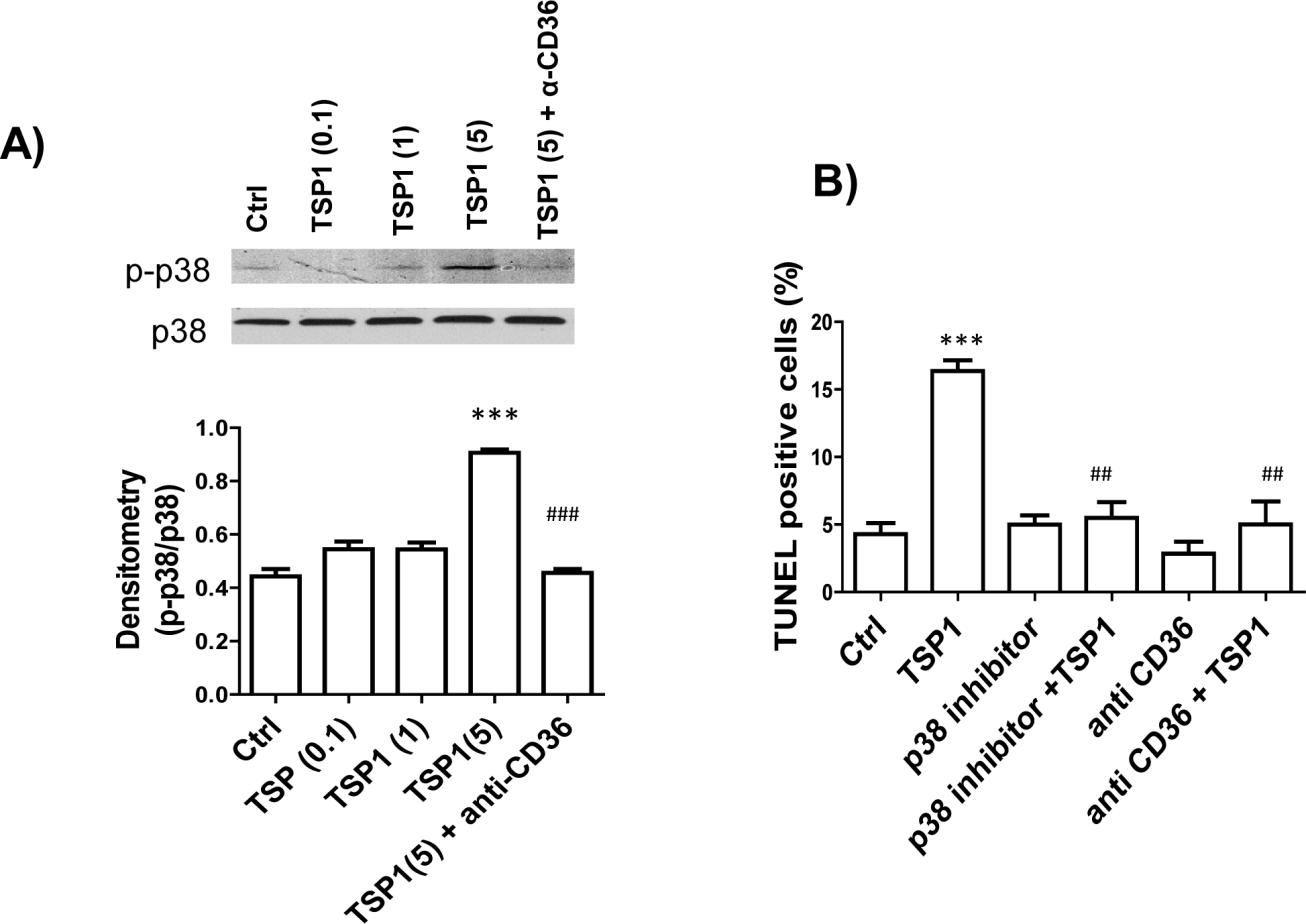
TSP1 treatment induced human podocyte apoptosis through activation of CD36-dependent p38 MAPK pathway. (A). Human podocytes were treated with different concentration of TSP1 (0.1, 1, and 5μg/ml) in the presence or absence of anti-CD36 antibody (15 μg/ml) for 15 minutes. Then phospho-p38MAPK level in cell lysates was determined by immunoblotting and normalized to total p38 levels. (B) Human podocytes were pre-incubated with SB202190 (p38 inhibitor, at 10^−5^ mol/L) and anti-CD36 antibody (15 μg/ml) for 2 h and then treated with TSP1 (5μg/ml) for 24 hours. Cells were then stained with TUNEL to calculate apoptotic cells. Data are presented as mean ± SE (n = 3 individual experiments). ***, *P* <0.001 vs. Ctrl; ##, P<0.01 vs.TSP1; ###, P<0.001 vs. TSP1 (5).

The mechanism of TSP1 induced podocyte apoptosis was further investigated. Previously, we demonstrated that interaction of TSP1 and CD36 contributes to obesity-associated podocytopathy [25]. CD36 has been shown to activate p38 MAPK pathway to mediate apoptosis induced by TSP1 in endothelial cells [37]. In platelets, CD36-dependent pathways including JNK and p38MAPK were activated by TSP1 [38]. Based on these reports, we determined whether activation of p38MAPK pathway is involved in TSP1-induced podocyte apoptosis. As shown in **Fig 4A**, we found that short time treatment of podocytes with TSP1 at 5 μg/ml stimulated phospho-p38 MAPRK levels, which was inhibited by anti-CD36 functional blocking antibody [39–41]. Furthermore, treatment of podocytes with an inhibitor of p38MAPK-SB212090 prevented TSP1 induced podocyte apoptosis (**Fig 4B),** suggesting that TSP1 induced podocyte injury was mediated by CD36-dependent activation of p38MAPK pathway.

### TSP1 deficient podocytes had reduced ADR-induced injury

**Fig 5:**
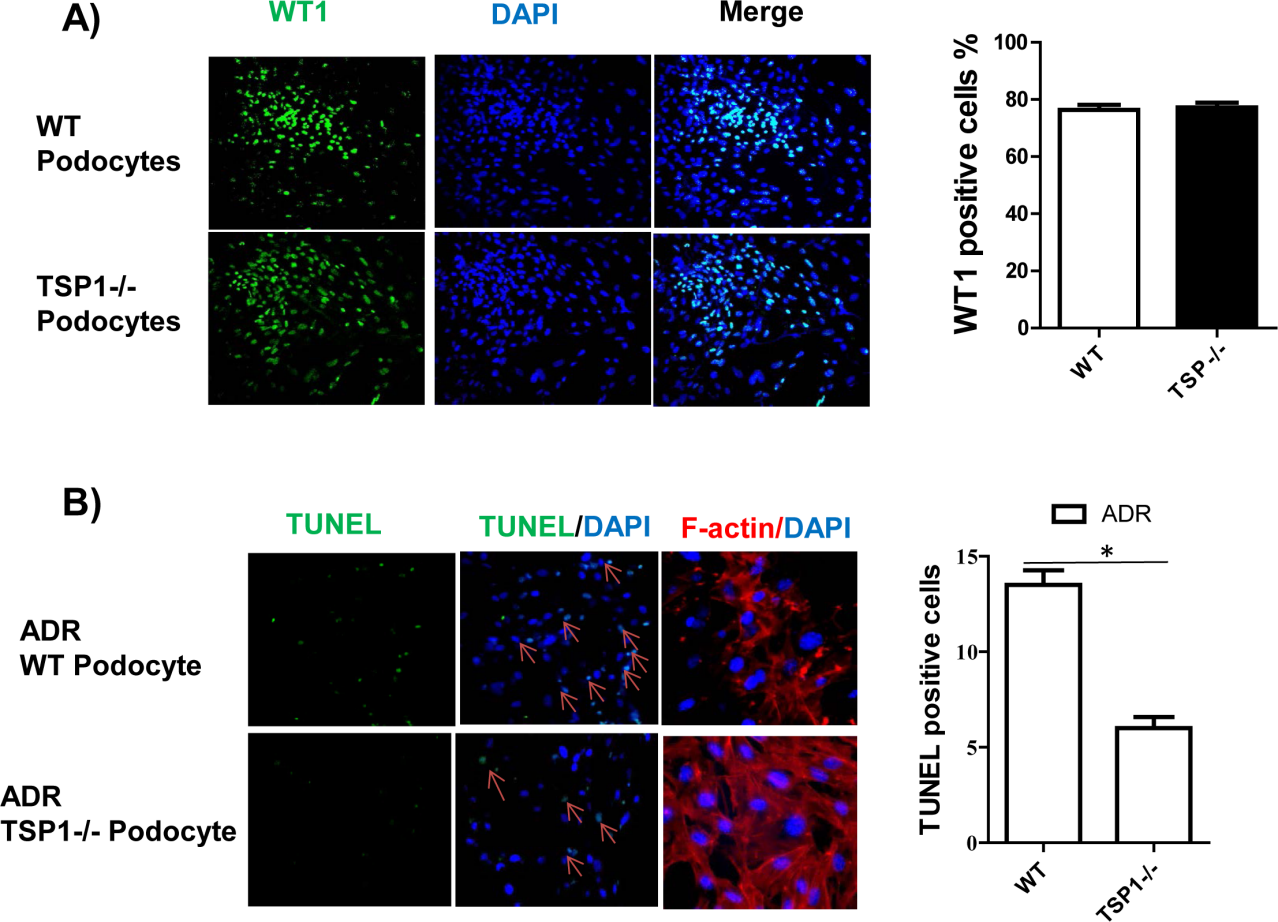
ADR induced podocyte apoptosis and actin cytoskeleton disorganization was attenuated in TSP1 deficient podocytes. (A). Podocytes were isolated from wild type or TSP1deficient mice and characterized by staining cells with WT1. (B). Cells were treated with ADR (20 μg/ml) for 24 h. After treatment, TUNEL staining and F-actin staining were performed. TUNEL positive cells were counted. Data are presented as mean ± SE (n = 3 individual experiments). *P<0.05.

To further determine the role of TSP1 in ADR induced podocyte injury, podocytes from TSP1 deficient mice and wild type control mice were isolated and characterized as previously described [25,35,36]. The purity of these cells was reached about 80% (**Fig 5A**). These cells were treated with ADR and then cell apoptosis or actin cytoskeletal disorganization was determined. The results demonstrated that ADR induced podocyte apoptosis and actin cytoskeleton disorganization was significantly diminished in TSP1 deficient podocytes (**Fig 5B**), suggesting that TSP1 partially mediates ADR induced podocyte injury.

### Genetic TSP1 deficiency attenuates ADR-induced podocyte injury and the development of proteinuric kidney disease

**Fig 6:**
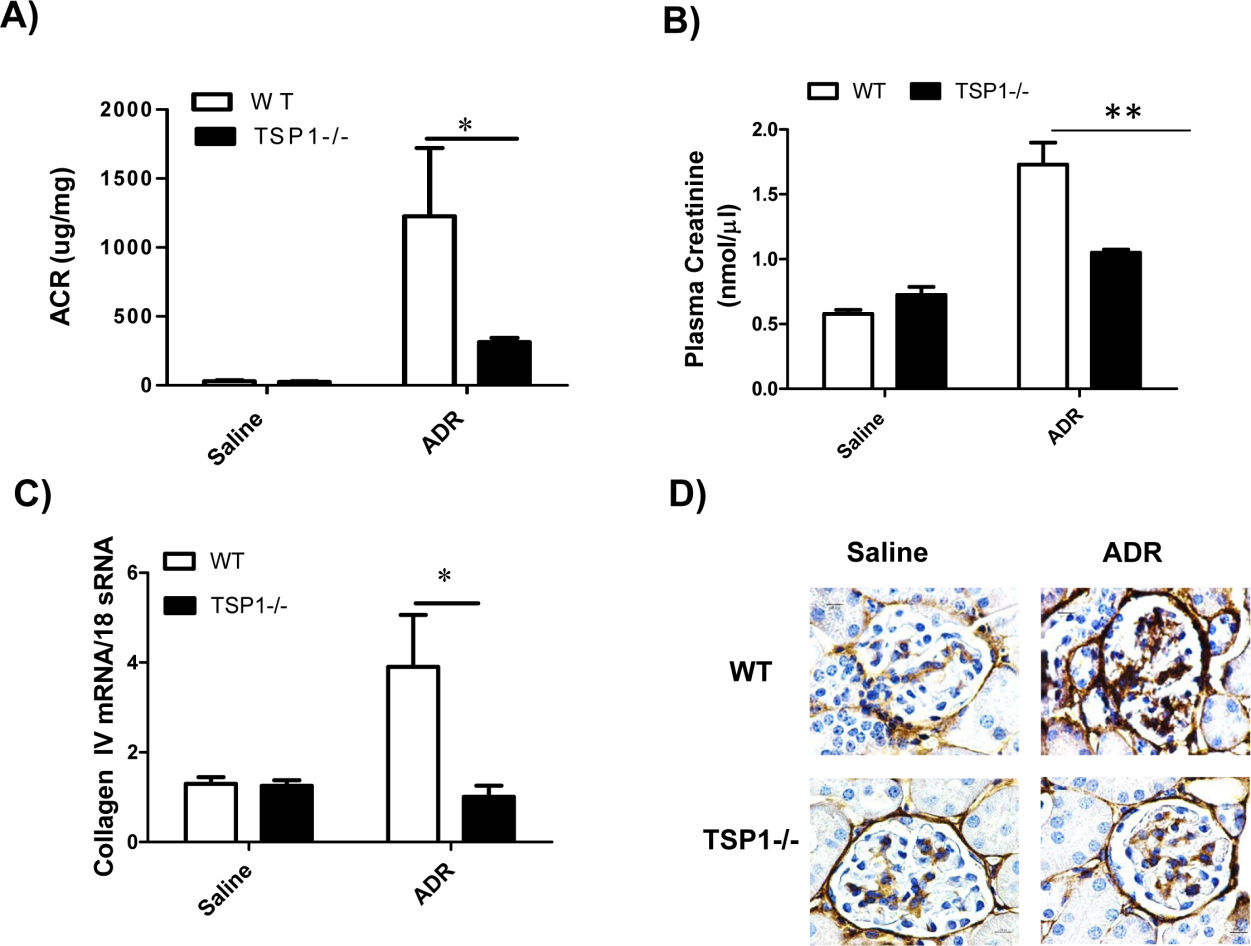
ADR-induced proteinuria and glomerulosclerosis was attenuated in TSP1 deficient mice. ADR was injected into male TSP1-/- mice and wild type control mice. After 14 days of injection, urinary albumin and creatinine ratio (A) and serum creatinine (B) levels were determined. (C) Glomerular collagen type IV mRNA levels were determined by real-time PCR. Data are presented as mean ± SE (n = 3–5 mice /group). *, *P* <0.05 and **, P<0.01. (D) Representative images of immunohistochemical staining of kidney samples with anti-collagen IV antibody were shown. The positive staining was shown as brown.

**Fig 7:**
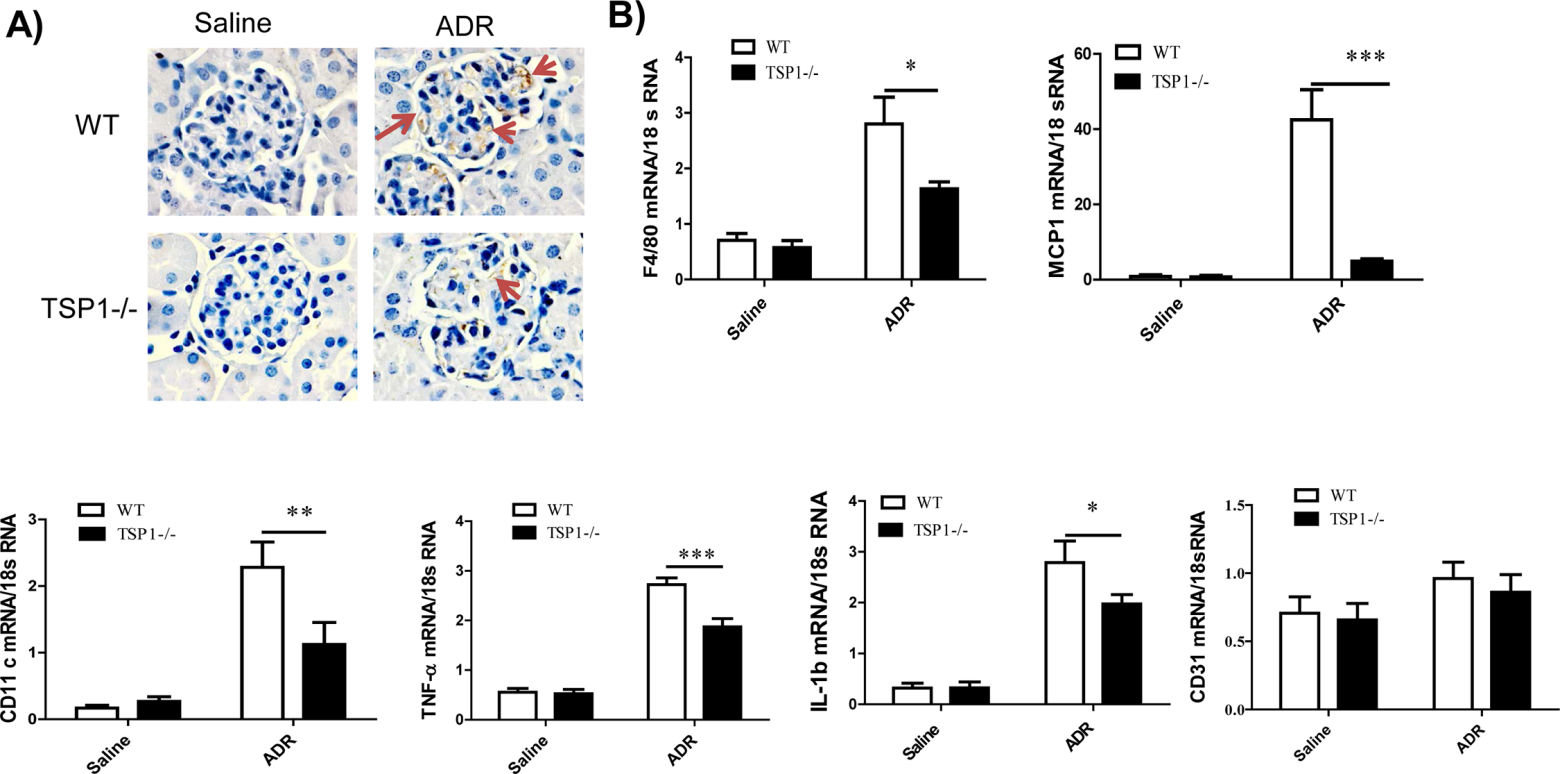
ADR-induced renal macrophage infiltration and inflammation was attenuated in TSP1 deficient mice. (A). Kidney samples from ADR or saline injected WT or TSP1-/- mice were stained with anti-F4/80 antibody. Representative images were shown. The positive brown staining was indicated by red arrows. (B). mRNA levels of pro-inflammatory cytokines including F4/80, MCP-1, CD11c, TNF-α, IL-1β and CD31 in the glomeruli were determined by real-time PCR. Data are presented as mean ± SE (n = 3–5 mice /group). * *P* <0.05; ** P<0.01; ***P<0.001.

**Fig 8:**
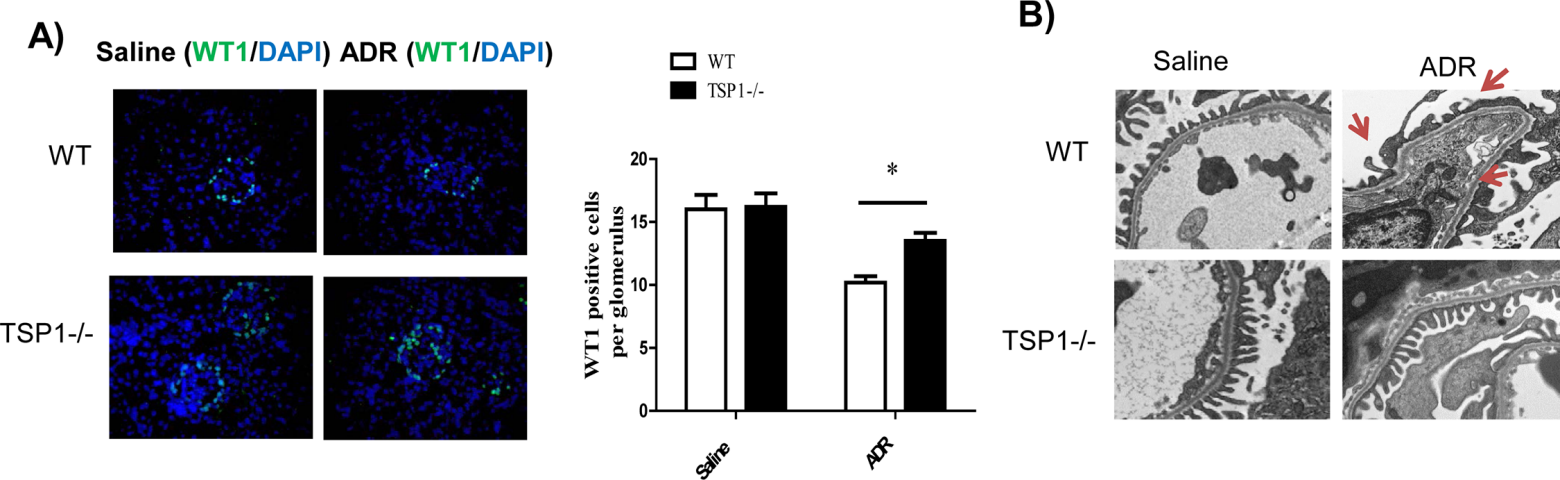
ADR-induced podocyte injury was attenuated in TSP1 deficient mice. ADR was injected into male TSP1-/- mice and wild type control mice to induced nephropathy. (A) Representative overlay images of immunofluorescence staining of frozen kidney sections with WT1 (green) and DAPI (purple) were shown. WT1 positive cells in glomeruli were counted. Data are presented as mean ± SE (n = 3–5 mice /group). *, *P* <0.05. (B) Representative images of electron microscopy showed that ADR injected wild type mice had foot process effacement (red arrow).

To determine whether TSP1 contributes to ADR-induced proteinuric kidney disease *in vivo*, global TSP1-/- mice and wild type controls were administered with ADR. The development of podocyte injury and proteinuria kidney disease in these mice was examined. At the end of the study, ADR administration induced similar level of body weight reduction in wild type and TSP1-/- mice (data not shown). In wild type mice, ADR injection induced proteinuria, renal fibrosis, and increased macrophage infiltration into glomeruli (q-PCR and immnohistochemical staining of F4/80 in kidney sections) and increased production of pro-inflammatory cytokines (q-PCR for MCP-1, CD11c, TNF-α, and IL-1β). All of these phenotypes were significantly reduced in TSP1-/- mice (**Figs 6 and 7**). In addition, angiogenesis marker-CD31 gene expression was comparable between wild type and TSP1-/- mice under either saline or ADR injection conditions (Fig 7B). Moreover, as compared to ADR injected wild type mice, ADR injected TSP1-/- mice had less podocyte loss (WT1 staining and counting in kidney sections) and podocyte foot process enfacement (from transmission electron microscopy images) (**Fig 8**). Taken together, these data provide *in vivo* evidence of the contribution of TSP1 to podocyte injury and the development of proteinuric kidney disease.

## Discussion

In the current study, we demonstrated that glomerular/podocytes TSP1 expression was upregulated in adriamycin (ADR) induced focal segmental glomerulosclerosis (FSGS) mouse model. ADR treatment also stimulated TSP1 expression in human podocytes *in vitro*. Increased TSP1 partially mediated ADR-induced podocyte apoptosis and actin cytoskeleton disorganization. This TSP1’s effect was through a CD36-dependent activation of p38MAPK pathway. Importantly, *in vivo* data demonstrated that TSP1 deficiency attenuated ADR induced nephropathy by showing attenuated proteinuria, reduced glomerulosclerosis, renal macrophage infiltration and inflammation, and podocyte injury. Taken together, these studies provide novel evidence of the contribution of TSP1 to podocyte injury and the development of non-diabetic proteinuric kidney disease.

TSP1 is a matricellular protein and expressed by many cell types including mesangial cells and podocytes. Accumulating evidence suggests that TSP1 is an important player in a variety of kidney diseases such as ischemia-reperfusion induced kidney injury and obesity/diabetes associate nephropathy [8–15,25]. Furthermore, current study provides new knowledge to the role of TSP1 in regulating podocyte function and its contribution to non-diabetic proteinuric kidney disease. In this study, we utilized a well-established podocyte injury and experimental FSGS model- adriamycin (ADR) induced nephropathy mouse model [30,31]. We found that glomeruli TSP1 was up-regulated from ADR injected mice and was mostly co-localized with podocytes. In patients with primary FSGS, increased TSP1 (mRNA and protein) was also found in podocytes of the sclerotic segments [24], suggesting that podocyte might be an important cellular source of increased TSP1 in glomeruli from both human and animal FSGS models. This is supported by our *in vitro* data showing that ADR stimulated TSP1 expression in cultured human podocytes but not in cultured mesangial cells or glomeruli endothelial cells. The stimulatory effect of ADR on TSP1 expression might be cell type specific and the detail molecular mechanism by which ADR stimulates TSP1 expression in podocytes warrants further investigation.

Increased TSP1 induces podocyte injury. Previously, we showed that addition of purified TSP1 to the cell culture media induced human podocyte to undergo apoptosis [25]. This effect has been confirmed in the current studies. Although TSP1 is an important regulator for latent TGF-β activation [42] and TGF-β induces podocyte apoptosis [43,44], we have demonstrated that TSP1 induced podocyte apoptosis is through a TGF-β independent mechanism [25]. In the current studies, we further determined the mechanisms of TSP1 induced podocyte apoptosis. TSP1 is a large homotrimer with multiple domains to interact with a variety of different cell surface receptors e.g. CD36, CD47, integrins. As a receptor for TSP1, CD36 exists on podocytes [45–47] and our current data demonstrated that CD36 expression was upregulated by ADR treatment *in vitro* as well as *in vivo*. Consistently, CD36 on podocytes was also upregulated in puromycin aminonucleoside induced rat FSGS [47]. Since CD36 mediates apoptotic signaling induced by TSP1 in endothelial cells [37], by ox-LDL in macrophages [48], by advanced end product (AGE) in tubular cells, or by palmitate in podocytes [25,45], we determined whether CD36 is involved in TSP1 mediated podocyte apoptosis. Our data demonstrated that TSP1 treatment stimulated phospho-p38MAPK pathway in podocytes, which was inhibited by pretreatment of anti-CD36 antibody. AKT of JNK pathway was not affected by TSP 1 treatment (data not shown). Moreover, an inhibitor of p38 MAPK prevented TSP1 induced podocyte apoptosis. Taken together, these data provide new evidence that TSP1 induces podocyte apoptosis is through a CD36 dependent mechanism and involves in stimulation of p38MAPK pathway. Our current studies demonstrated a contribution of extracellular interaction of TSP1 with its receptor to podocyte injury. Recently, TSP1 intracellular interactions have been reported. Interestedly, an interaction of TSP1 with ERK in the cytoplasm in Ras retrovirus infected lung fibroblasts has been shown by Bake et al [49]. Whether this TSP1 intracellular interaction contributes to podocyte apoptosis in FSGS model is unknown and will be determined in the future.

In addition to the direct effect of TSP1 on podocyte injury, our studies also suggest a pro-inflammatory effect of TSP1 on podocyte injury and the development of proteinuric kidney disease. It is known that inflammation is an important causal factor in the development of chronic kidney disease. Several components of innate immunity including TLRs and macrophages have been implicated in the progression of renal disease [50]. Our previous studies have demonstrated that TSP1 is an important regulator of macrophage function and plays a role in obesity associated inflammation and insulin resistance [51]. A pro-inflammatory role of TSP1 in the development of chronic kidney disease (ureteric obstruction model) has been also reported [52]. In agreement with these reports, in the current study, we found that ADR administered TSP1 deficient mice had reduced renal MCP-1 level, which was associated with reduced macrophage infiltration into the kidney. The expression of pro-inflammatory cytokines such as TNF-α and IL-1β in the kidney was also significantly reduced in these mice. Together, these data suggest a role for TSP1 in promoting renal inflammation in FSGS mouse model. Since TSP1 is an anti-angiogenesis factor, we also determined whether TSP1 regulated angiogenesis and contributed to ADR-nephropathy. We found that kidney CD31 mRNA levels were comparable among WT saline, TSP1-/- saline, WT ADR and TSP1-/- ADR mice (Fig 7B). This data suggests that angiogenesis is unlikely to play a role in TSP1 mediated proteinuria kidney injury in ADR-model.

In summary, our data demonstrated that TSP1 is an important contributor to podocyte injury and the development of non-diabetic proteinuric kidney disease. The effect of TSP1 on podocyte injury is mediated by a CD36-dependent activation of p38MAPK pathway. TSP1 also stimulates macrophage infiltration and renal inflammation in FSGS model. These data may provide new therapeutic strategies for treatment of proteinuric kidney disease.
